# Supplementary material for: Biochemical and functional characterization of mutant KRAS epitopes validates this oncoprotein for immunological targeting
Source: Nat Commun. 2021 Jul 16;12:4365. doi: 10.1038/s41467-021-24562-2 (PMC8285372; doi:10.1038/s41467-021-24562-2)
Supplement: Supplementary file 1 — Supplementary Information [file 41467_2021_24562_MOESM1_ESM.pdf]

Supplementary Information for:

Biochemical and Functional Characterization of Mutant KRAS Epitopes Validates this Oncoprotein for Immunological Targeting

Authors

Adham S. Bear[1,2]*, Tatiana Blanchard[3], Joseph Cesare[4], Michael J. Ford[5], Lee P. Richman[2], Chong Xu[3], Miren L. Baroja[3], Sarah McCuaig[6], Christina Costeas[6], Khatuna Gabunia[3], John Scholler[3], Avery D. Posey Jr.[3,7,8], Mark H. O'Hara[1,2], Anze Smole[3], Daniel J. Powell Jr.[3,9], Benjamin A. Garcia[10], Robert H. Vonderheide[2,3,11§], Gerald P. Linette[1,3,11§], Beatriz M. Carreno[3,9,11§]*

Affiliations:

[1] Division of Hematology-Oncology, Department of Medicine, Perelman School of Medicine, University of Pennsylvania, Philadelphia, PA USA
[2] Abramson Cancer Center, University of Pennsylvania, Philadelphia, PA USA
[3] Center for Cellular Immunotherapies, Perelman School of Medicine, University of Pennsylvania, Philadelphia, PA USA
[4] Department of Biochemistry and Biophysics, University of Pennsylvania, Philadelphia, PA USA
[5] MSBioworks, Ann Arbor, MI, USA
[6] Perelman School of Medicine, University of Pennsylvania, Philadelphia, PA USA
[7] Department of Systems Pharmacology and Translational Therapeutics, Perelman School of Medicine, University of Pennsylvania, Philadelphia, PA USA
[8]Corporal Michael J. Crescenz VA Medical Center, Philadelphia, PA, USA
[9] Department of Pathology and Laboratory Medicine, Perelman School of Medicine, University of Pennsylvania, Philadelphia, PA USA
[10] Epigenetics Institute, Department of Biochemistry and Biophysics, University of Pennsylvania, Philadelphia, PA USA
[11] Parker Institute for Cancer Immunotherapy, Perelman School of Medicine, University of Pennsylvania, Philadelphia, PA USA

[§]These authors jointly supervised this work

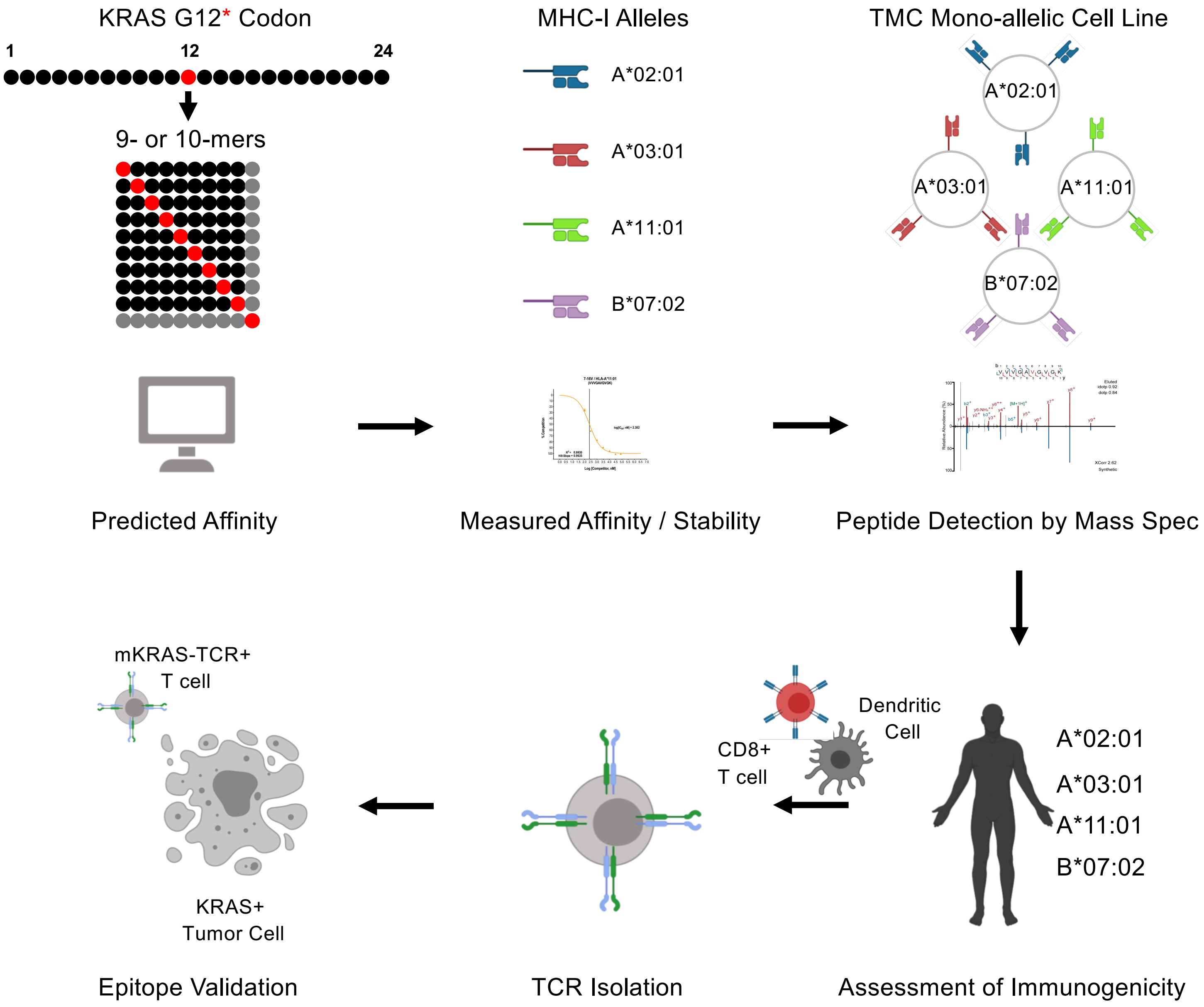

Supplementary Figure 1: Mutant KRAS epitope discovery and validation pipeline. Schematic diagram showing computational, biochemical, proteomic and immunological assays used for mKRAS epitope identification. Computational neoantigen prediction was used to identify HLA class I-restricted 9- and 10-mer WT and mKRAS G12 epitope candidates. Biochemical assays were performed to characterize mKRAS p-HLA affinity and stability. Processing and presentation of mKRAS neoantigens was investigated by HLA class I immunoprecipitation, peptide elution and tandem / targeted mass spectrometry using monoallelic cell lines expressing a tandem minigene construct (TMC). Immunogenicity was examined using purified $CD8^+$ T cells from healthy donors stimulated twice with autologous mature dendritic cells pulsed with peptides followed by assessment of antigen specificity. mKRAS epitopes were validated by the isolation of mKRAS-specific TCRs which were used as probes to detect tumor cell antigen processing and presentation. This figure was created with BioRender.com.

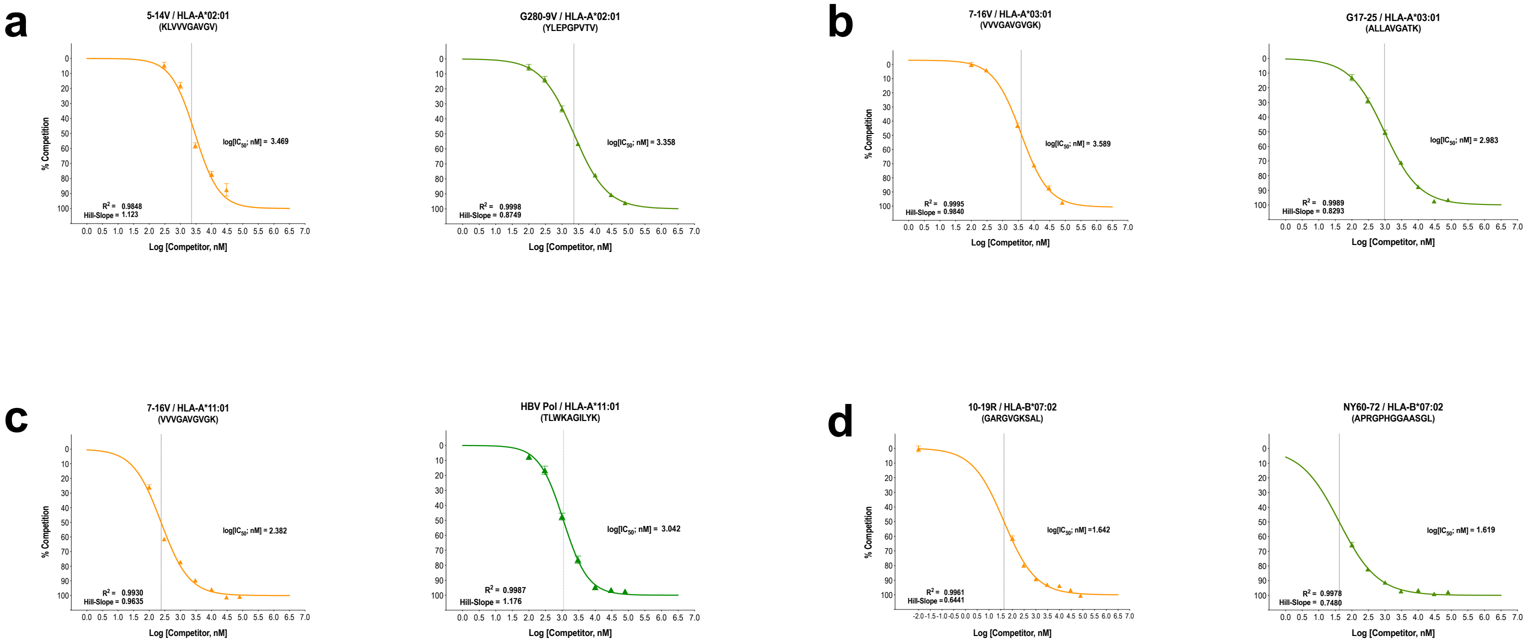

Supplementary Figure 2: Experimental measurement of mKRAS epitope affinities by fluorescence polarization competitive peptide binding assays. Soluble HLA-class I competition assays testing titrated concentrations of unlabeled test peptide for competition of FITC-labeled reference peptide. Representative examples of results obtained with candidate mKRAS peptides (left panels) and corresponding positive peptide controls (right panels) are shown for **(a)** HLA-A*02:01, **(b)** HLA-A*03:01, **(c)** HLA-A*11:01, and **(d)** HLA-B*07:02. Data are presented as mean values +/- SD. The IC_{50} values of test peptides is determined by fitting data to dose-response model using Prism software, and the $\log_{10}[IC_{50}(nM)]$ values are reported as per published method.[1] R^2 indicates the goodness of fit. Peptides are classified as high affinity ($\log_{10}[IC_{50}]$<3.7nM), medium affinity ($\log_{10}[IC_{50}]$=3.7-4.7nM), low affinity ($\log_{10}[IC_{50}]$=4.7-5.5nM) and very low affinity ($\log_{10}[IC_{50}]$=5.5-6.0nM). Data are representative of 2 independent experiments. Source data are provided as a Source Data file.

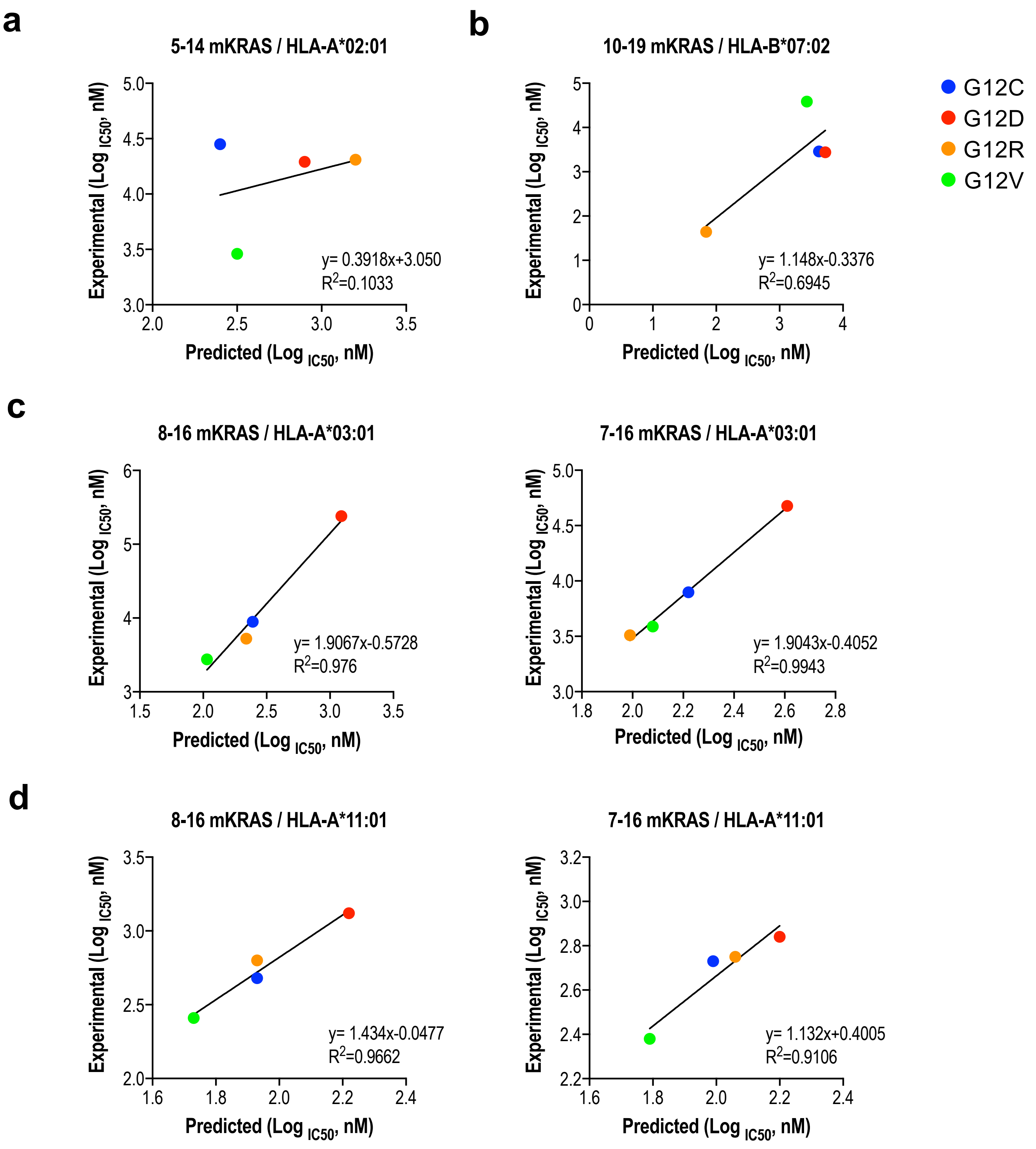

Supplementary Figure 3: Predicted vs experimental affinity comparison of candidate mKRAS epitopes. To evaluate the relationship between predicted (antigen.garnish) and experimentally (fluorescence polarization) determined peptide affinities, the $\log_{10}[IC_{50}(nM)]$ values were graphed against each other and a correlation factor was determined by linear regression analysis with R^2 indicating goodness of fit. **(a)** Predicted vs experimental affinity of 5-14 mKRAS peptides to HLA-A*02:01. **(b)** Predicted vs experimental affinity of 10-19 mKRAS peptides to HLA-B*07:02. **(c)** Predicted vs experimental affinity of 8-16 and 7-16 mKRAS peptides to HLA-A*03:01. **(d)** Predicted vs experimental affinity of 8-16 and 7-16 mKRAS peptides to HLA-A*11:01. For each class I allele, 4 peptides were evaluated as indicated in the figure legend.

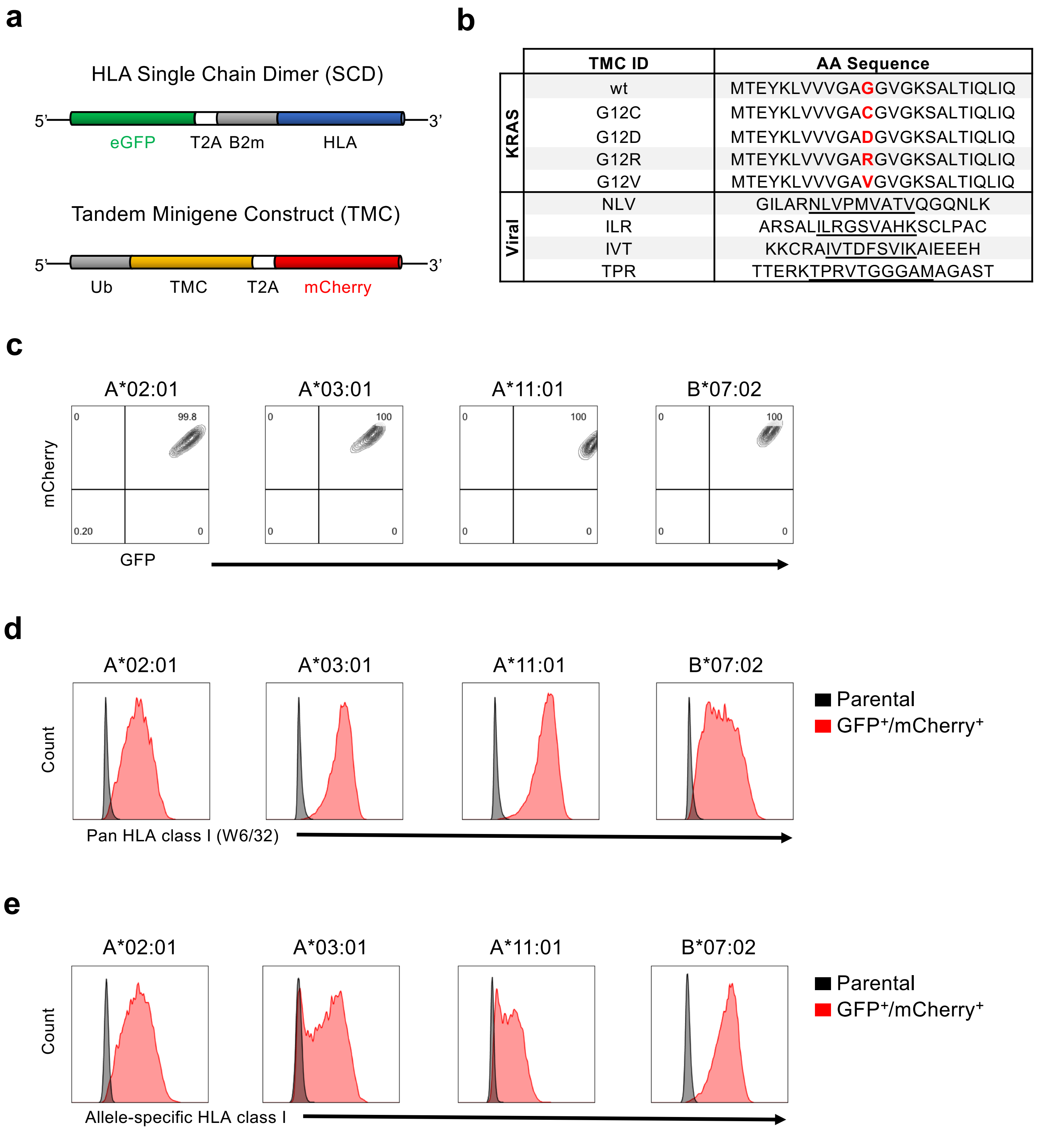

Supplementary Figure 4: Generation of single-allele (monoallelic) HLA class I / tandem minigene construct-expressing cell lines. **(a)** Design of HLA-SCD and TMC lentiviral vector constructs. **(b)** Table displaying minigene-encoded amino acid sequences of WT and mKRAS 25-mer peptides as well as validated viral epitopes of HLA-A*02:01 (NLV peptide), HLA-A*03:01 (ILR peptide), HLA-A*11:01 (IVT peptide) and HLA-B*07:02 (TPR peptide) included in TMC. **(c)** FACS plot demonstrating co-expression of HLA-SCD (eGFP) and TMC (mCherry) constructs in K562 cells following lentiviral transduction and flow cytometric cell sorting. **(d)** FACS plot demonstrating HLA class I expression as detected by pan antiboby W6/32 by $GFP^{+}mCherry^{+}$ transductants (*Red*) as compared to parental K562 cells (*Black*). **(e)** FACS plot confirming HLA class I allele-specific surface expression by $GFP^{+}mCherry^{+}$ transductants (*Red*) as compared to parental K562 cells (*Black*).

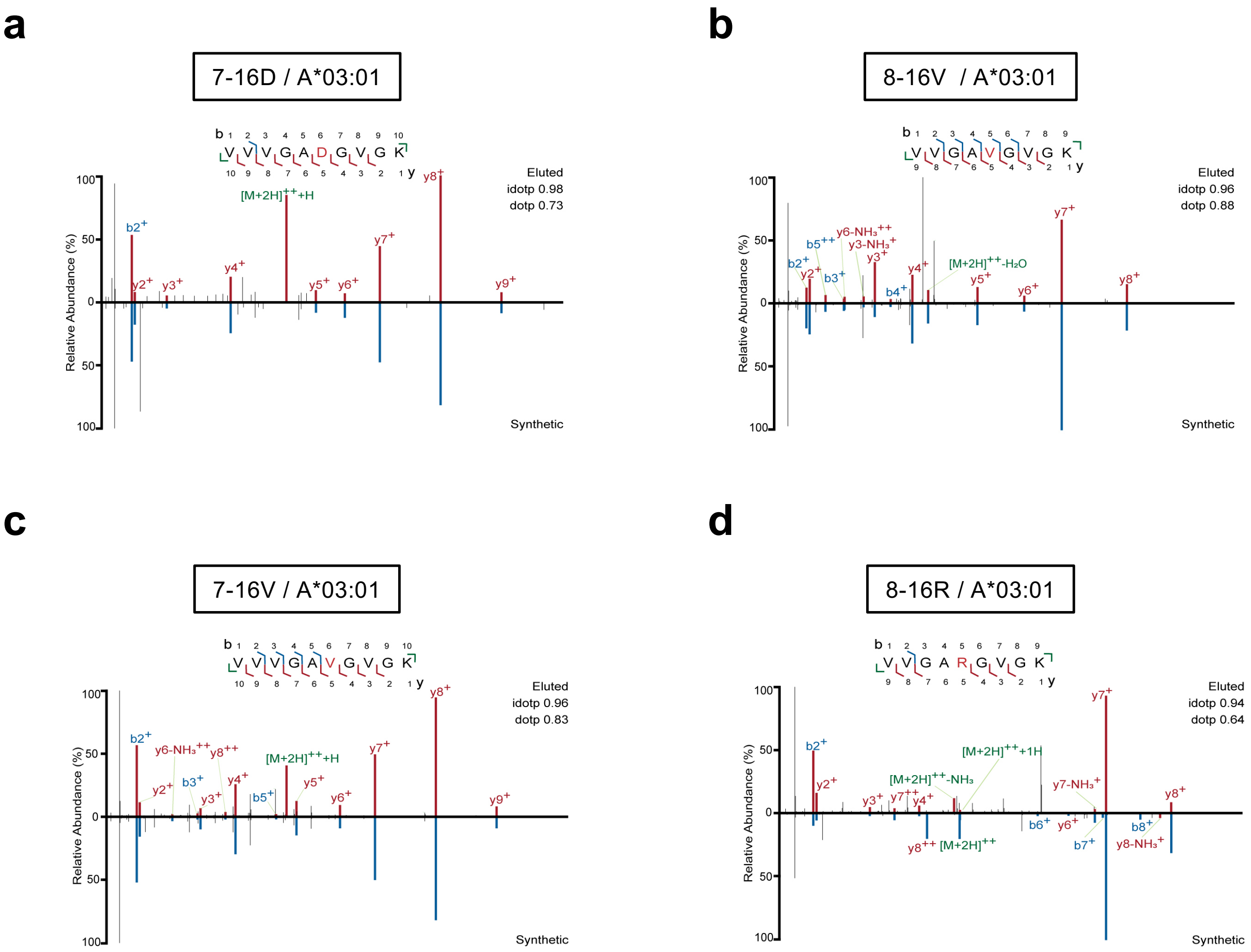

Supplementary Figure 5: Validation of HLA-A*03:01-restricted TMC-encoded mKRAS epitope processing and presentation. MS/MS fragmentation pattern of **(a)** 7-16D, **(b)** 8-16V, **(c)** 7-16V and **(d)** 8-16R ions eluted from HLA-A*03:01 expressed on monoallelic TMC-expressing cells (*Red Upper*) and the corresponding synthetic peptide (*Blue Lower*). For further AA sequence adjudication as calculated by Skyline,[2] values obtained for idotp (correlation of precursor isotope distribution in eluted vs. synthetic) and dotp (correlation of precursor isotope distribution in eluted vs. library spectrum in Proteome Discover Database) are provided.

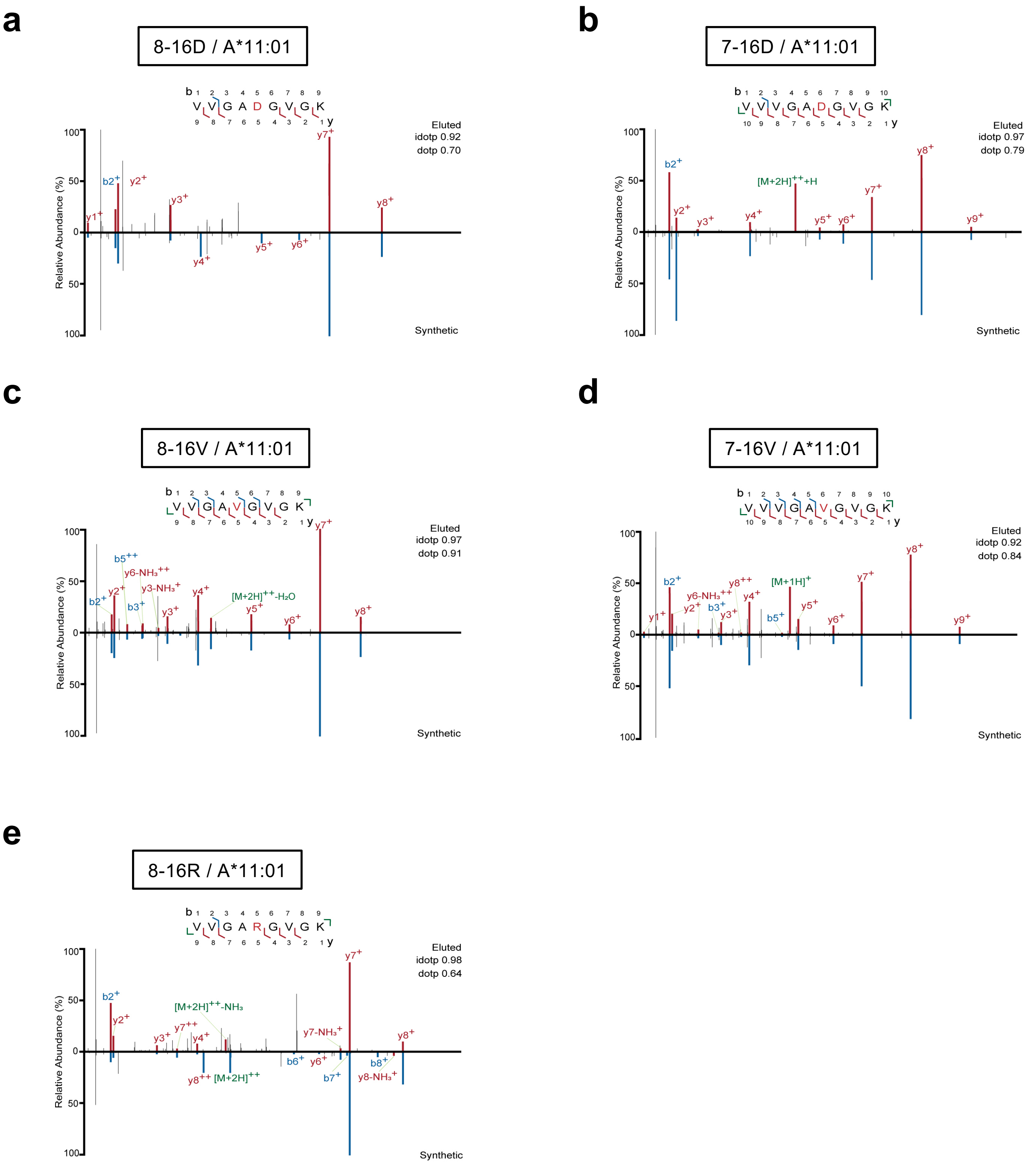

Supplementary Figure 6: Validation of HLA-A*11:01-restricted TMC-encoded mKRAS epitope processing and presentation. MS/MS fragmentation pattern of **(a)** 8-16D, **(b)** 7-16D, **(c)** 8-16V and **(d)** 7-16V and (e) 8-16R ions eluted from HLA-A*11:01 expressed on monoallelic TMC-expressing cells (*Red Upper*) and the corresponding synthetic peptide (*Blue Lower*). Idotp and dotp values are reported.

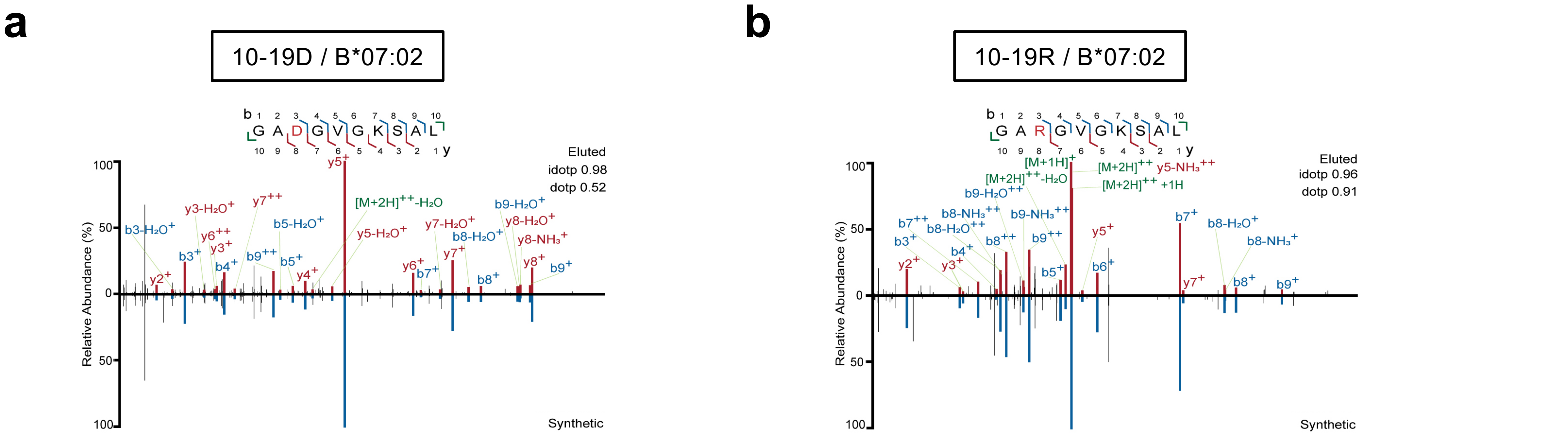

Supplementary Figure 7: Validation of HLA-B*07:02-restricted TMC-encoded mKRAS epitope processing and presentation. MS/MS fragmentation pattern of **(a)** 10-19D and **(b)** 10-19R ions eluted from HLA-B*07:02 expressed on monoallelic TMC-expressing cells (*Red Upper*) and the corresponding synthetic peptide (*Blue Lower*). Idotp and dotp values are reported.

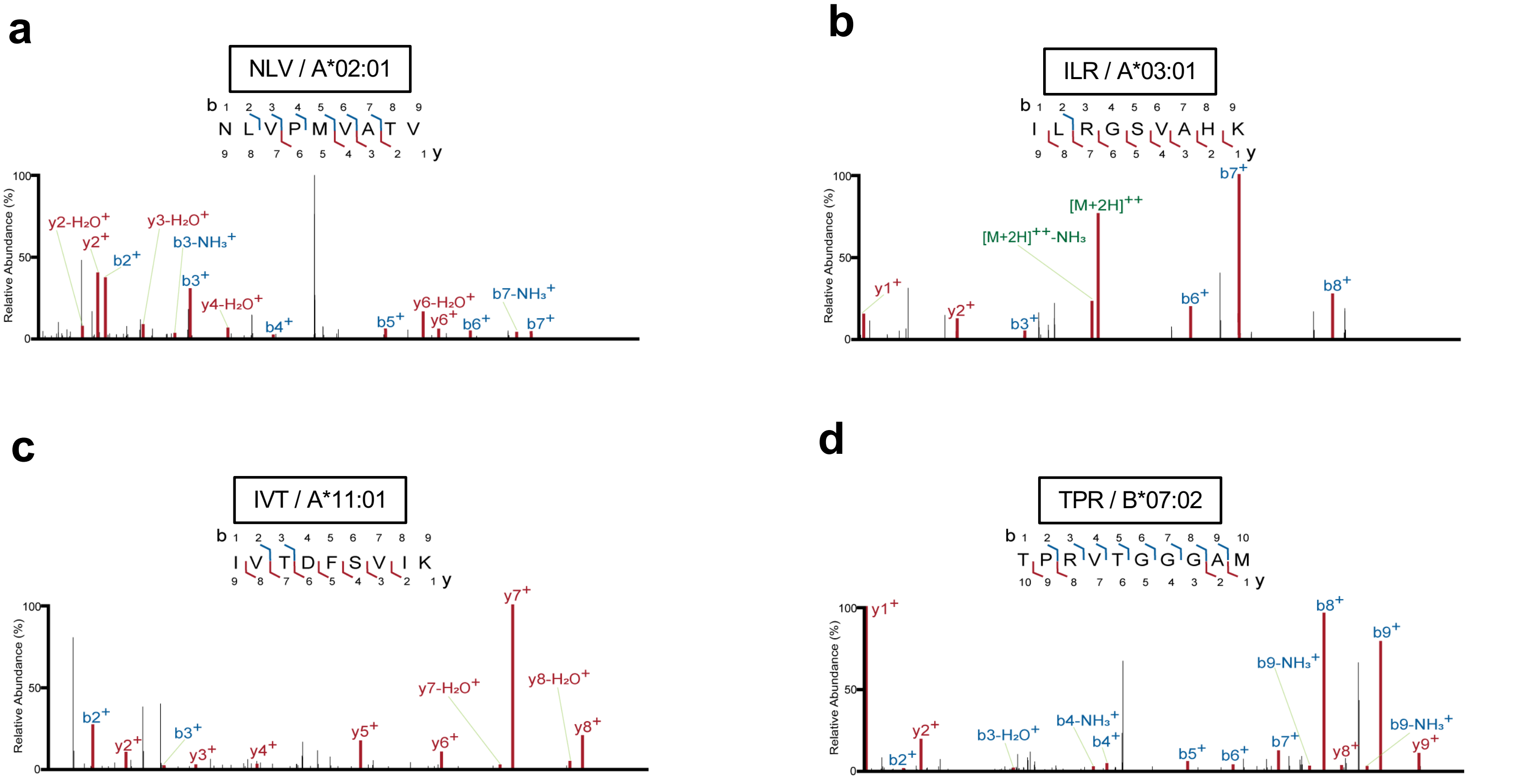

Supplementary Figure 8: Validation of TMC-encoded viral epitope processing and presentation. MS/MS fragmentation pattern of viral ions eluted from **(a)** HLA-A*02:01 (NLV peptide), **(b)** HLA-A*03:1 (ILR peptide), **(c)** HLA-A*11:01 (IVT peptide) and **(d)** HLA-B*07:02 (TPR peptide) expressed on monoallelic TMC-expressing cells.

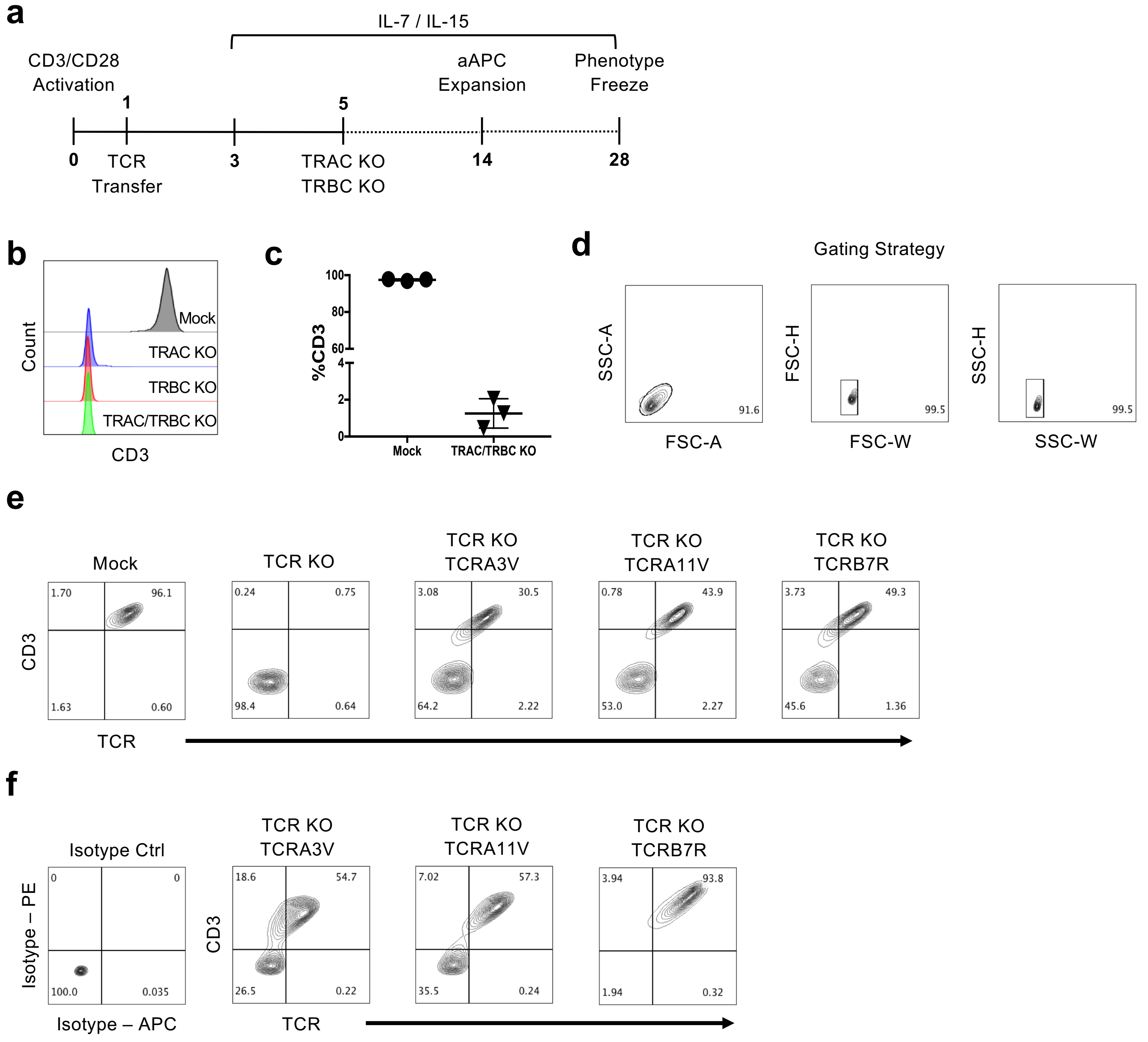

Supplementary Figure 9: mKRAS TCRαβ gene transfer to TCR gene-edited primary CD8⁺ T cells. (a) Experimental Timeline: Primary CD8⁺ T cells obtained from healthy donors were stimulated with CD3/CD28 microbeads. Activated CD8⁺ T cells were engineered with lentiviral particles (MOI = 5) encoding TCR constructs 16h later. Cells were cultured in the presence of IL-7 and IL-15 beginning on day 3. On day 5, CD3/CD28 microbeads were removed prior to CRISPR/Cas9-mediated TCRαβ gene-editing using sgRNAs targeting *TRAC* and *TRBC1/TRBC2* loci. Cells were expanded in culture for 14 days, phenotyped, and restimulated with peptide-pulsed monoallelic artificial APCs. Cells were expanded in culture for an additional 14 days, phenotyped, and frozen for future assays. **(b)** FACS histogram of CD3 expression on primary CD8⁺ T cells following CRISPR/Cas9-mediated *TRAC*, *TRBC* or *TRAC/TRBC* KO and 14-day in vitro expansion. **(c)** Frequency of CD3 expression on CD8⁺ T cells following CRISPR/Cas9-mediated *TRAC/TRBC* KO and 14-day in vitro expansion using primary CD8⁺ T cells from healthy donors (n=3). Data are presented as mean values +/- SD. **(d)** FACS plots demonstrating gating strategy used to collect viable single cell events utilized to generate the following manuscript figures: Figures 2, 3, 4 and Supplemental Figures 4, 9, 10, 12. **(e)** CD8⁺ T cell phenotype at day 14 following mKRAS-TCR gene transfer and *TRAC/TRBC* gene editing (TCR KO). Cells expressing mKRAS-TCRs retain CD3 and TCRαβ coexpression. Unmanipulated (Mock) and untransduced TCR KO CD8⁺ T cells (TCR KO) served as positive and negative staining controls, respectively. **(f)** CD3 and TCR expression in mKRAS TCR-engineered CD8⁺ T cell at day 28 following secondary aAPC expansion.

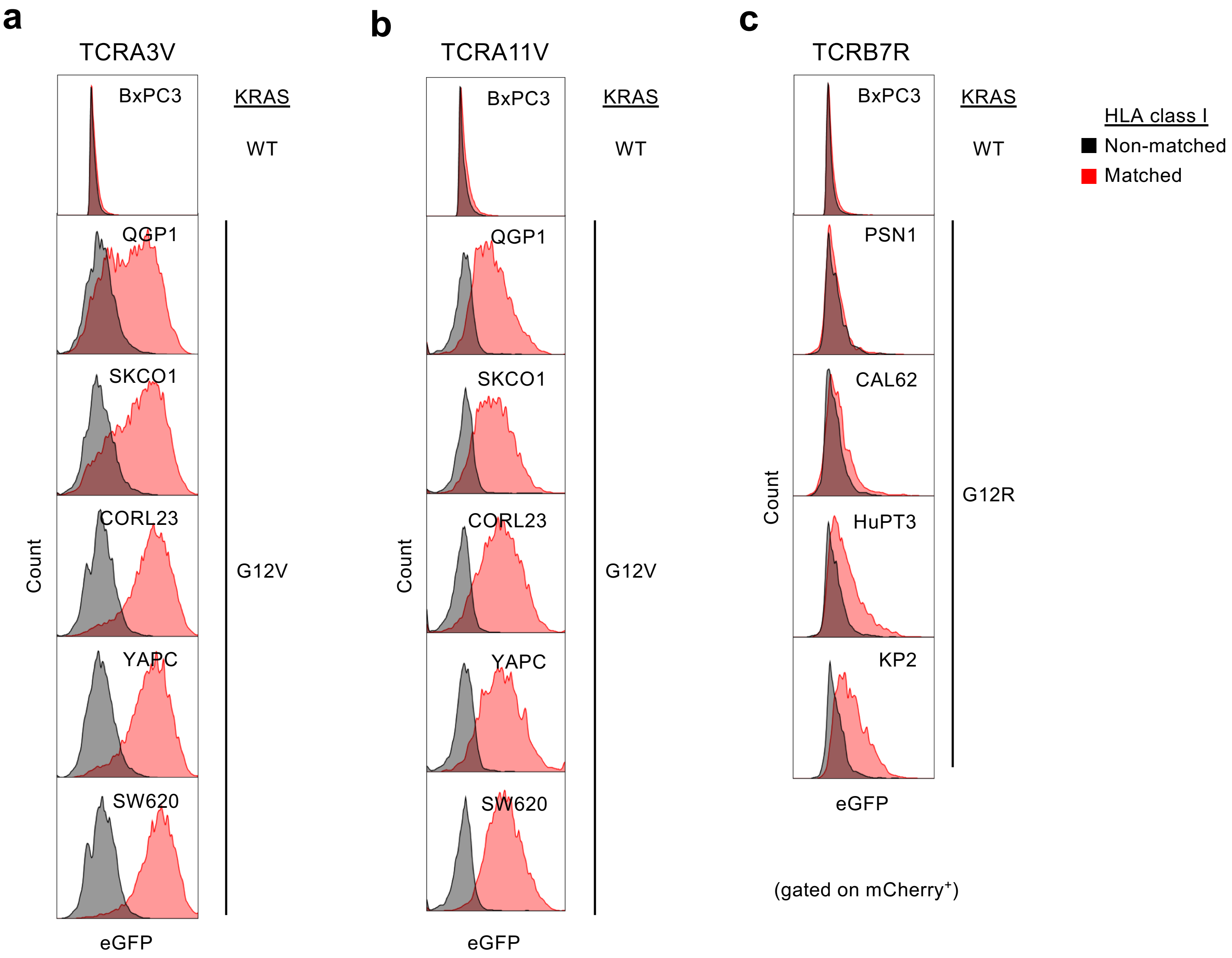

Supplementary Figure 10: mKRAS-TCR engineered J^{ASP90} reporter cells detect endogenous mKRAS epitopes presented by HLA-matched tumor cell lines. (a) FACS histogram plots demonstrating eGFP expression upon NFAT activation following 24h coculture of J^{ASP90} reporter cells expressing **(a)** TCRA3V or **(b)** TCRA11V with HLA-matched (*Red*) or non-matched (*Black*) KRAS WT (BxPC-3) or G12V^{+} tumor cell lines **(c)** FACS histogram plots demonstrating eGFP expression upon NFAT activation following 24h coculture of J^{ASP90} reporter cells expressing TCRB7R with HLA-matched (*Red*) or non-matched (*Black*) KRAS WT (BxPC-3) or G12R^{+} tumor cell lines pretreated with IFN-γ for 48h.

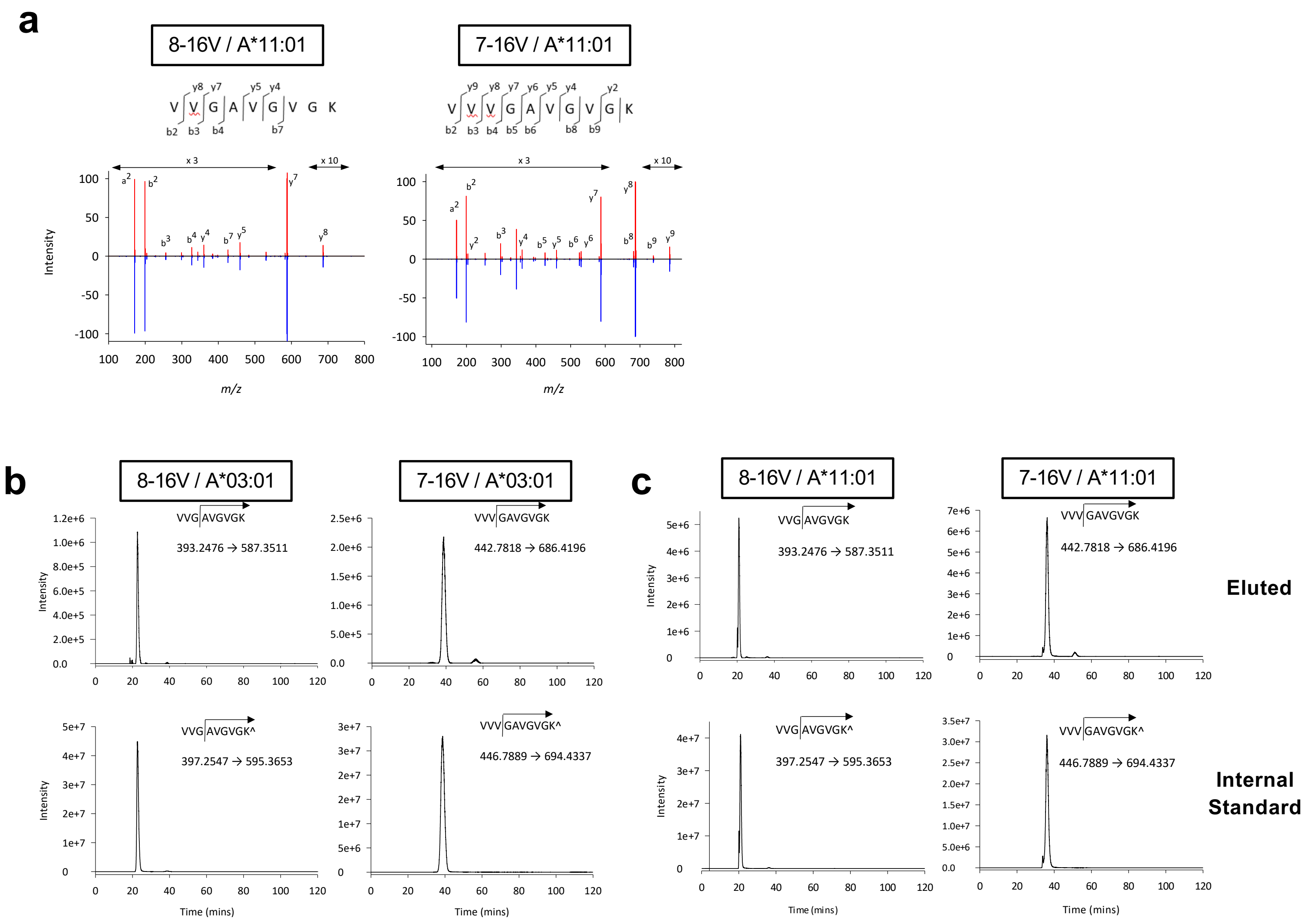

Supplementary Figure 11: Quantitation of HLA-A3- and HLA-A11-restricted KRAS G12V peptide ligands. (a) Representative MS/MS fragmentation pattern of 8-16V and 7-16V ions eluted from CORL23-A11 tumor cells (*Red Upper*) compared to synthetic peptide (*Blue Lower*). **(b)** Absolute quantification analysis of processed and presented 8-16V and 7-16V epitopes expressed by CORL23-A3 cells. LC-SRM traces for the specific parent-to-product ion transitions for eluted 8-16V and 7-16V (*Upper*) along with internal standard peptides (*Lower*) are shown. **(c)** Absolute quantification analysis of processed and presented 8-16V and 7-16V epitopes expressed by CORL23-A11 cells. LC-SRM traces for the specific parent-to-product ion transitions for eluted 8-16V and 7-16V (*Upper*) along with internal standard peptides (*Lower*) are shown. Internal standard peptides (100 fmol) have a C-terminal stable labeled lysine (K^) of chemical composition $^{13}C_6{}^{15}N_2$.

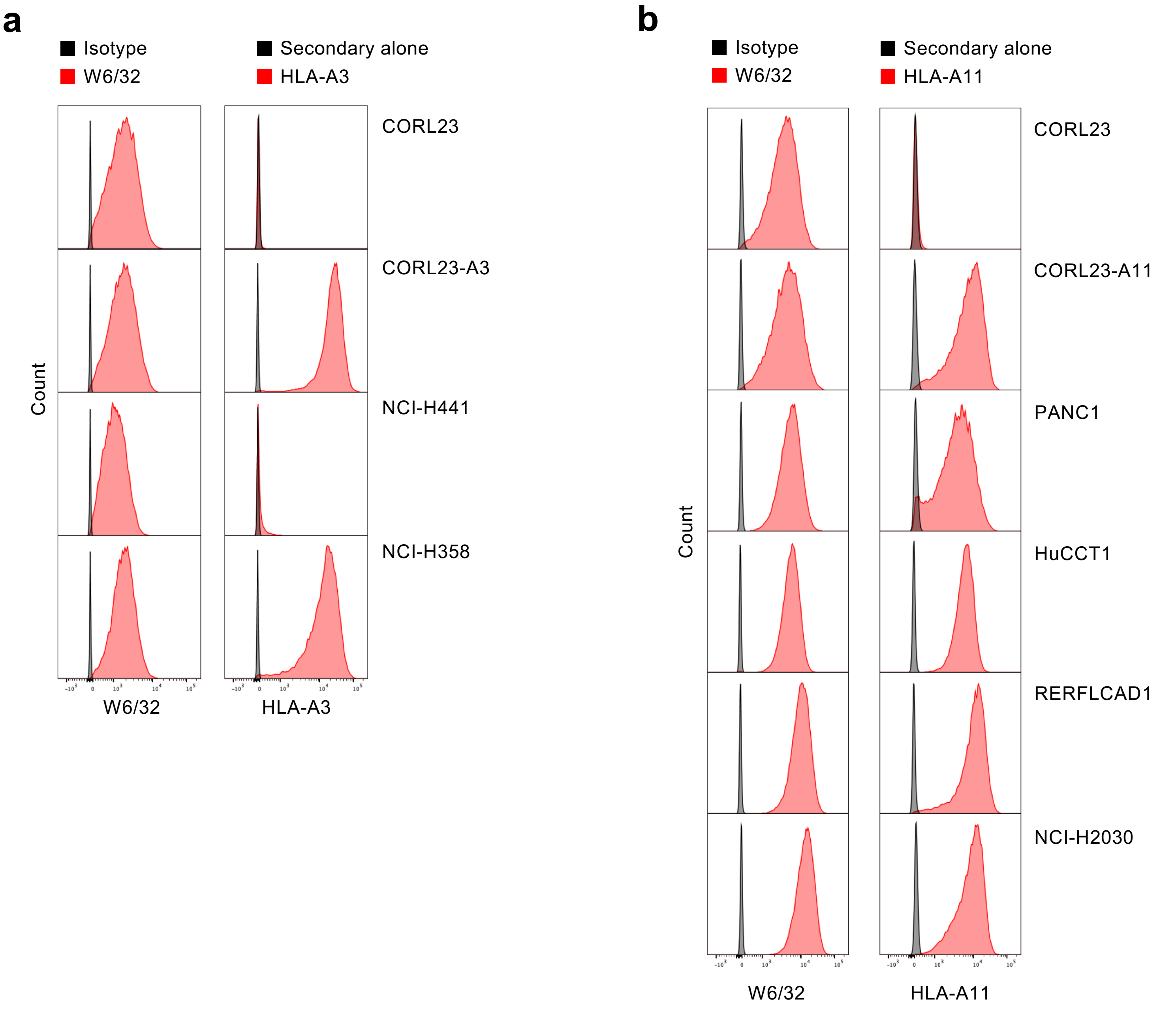

Supplementary Figure 12: HLA class I expression on parental and engineered tumor cell lines. (a) FACS histogram analysis to evaluate the expression of total HLA class I (left, as detected by W6/32) and HLA-A*03:01 (right, allele-specific Ab) molecules by CORL23-A3 as compared to non-engineered HLA-A*03:01^{+} NCI-H441 and NCI-H358 mKRAS tumor cell lines. **(b)** FACS histogram analysis to evaluate the expression of total HLA class I (left) and HLA-A*11:01 (right) molecules by CORL23-A11 cells as compared to non-engineered HLA-A*11:01^{+} mKRAS tumor cell lines. Parental COR-L23 are shown as HLA allele-specific controls for staining in (a) and (b). *Red* histograms are APC-conjugated W6/32 and APC-conjugated allele-specific stained samples, *Black* histograms represent staining controls APC-conjugated isotype antibody for W6/32 and APC-conjugated streptavidin for HLA allele-specific staining.

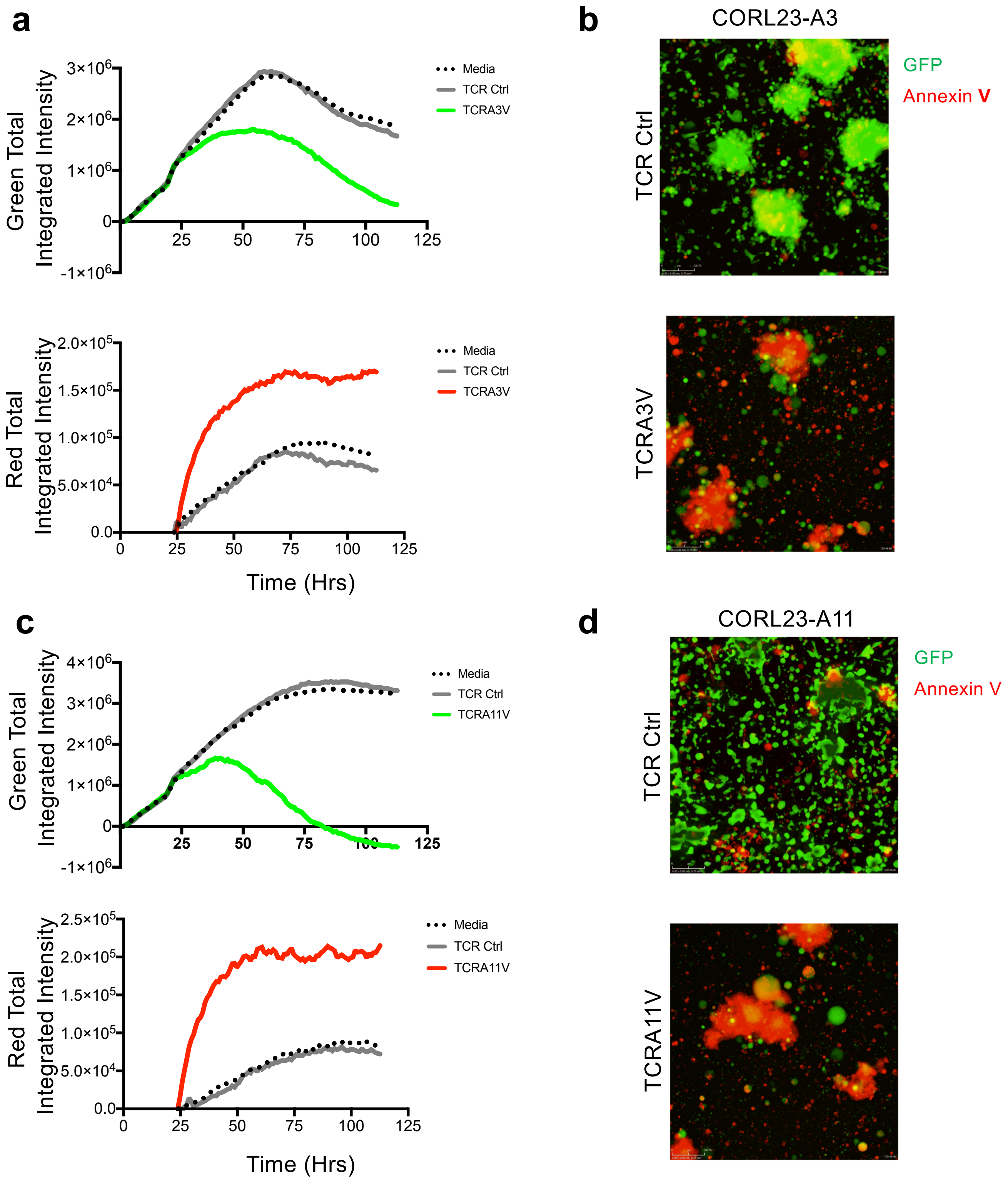

Supplementary Figure 13: Live cell imaging to assess TCRA3V and TCRA11V T cell cytotoxic activity against COR-L23 tumor lines. **(a)** Total Green (eGFP expressed by tumors) and Red (Annexin V-CF594) integrated intensity data of T cells cultured with CORL23-A3 cells. Colored lines correspond to wells with tumor cells cultured with TCRA3V cells compared to TCR Ctrl (TCRA11V) cells (*Black*) and media alone (*Dashed*). Data represent E:T ratio of 1:1. Data are presented as mean values (n=2). **(b)** Representative Green/Red overlay images at 110h comparing CORL23-A3 cells cultured with TCRA3V or TCR Ctrl cells. **(c)** Total Green and Red integrated intensity data of T cells cultured with CORL23-A11 cells. Colored lines correspond to wells with tumor cells cultured with TCRA11V cells compared to TCR Ctrl (TCRA3V) cells (*Black*) and media alone (*Dashed*). Data represent E:T ratio of 1:1. Data are presented as mean values (n=2). **(d)** Representative Green/Red overlay images at 110h comparing CORL23-A11 cells cultured with TCRA11V or TCR Ctrl cells. Data are representative of 2 independent experiments. Source data are provided as a Source Data file.

Supplementary Table 1: Predicted and experimental p-HLA binding affinities of candidate mKRAS G12 epitopes. Binding affinities were predicted using the *antigen.garnish* neoantigen prediction tool. Affinities are reported as IC_{50} (nM) and as $log_{10}[IC_{50}(nM)]$ for comparison to experimental values. Experimental affinities were determined by fluorescence polarization competition assays. Peptides are classified as high affinity ($log_{10}[IC_{50}]$<3.7nM), medium affinity ($log_{10}[IC_{50}]$=3.7-4.7nM), low affinity ($log_{10}[IC_{50}]$=4.7-5.5nM) and very low affinity ($log_{10}[IC_{50}]$=5.5-6.0). Peptide AA sequences are shown with AA at G12 codon position indicated in *Red*.

HLA Restriction	Peptide ID	G12 AA	Peptide Length	AA Sequence	Predicted Affinity (nM)		*Experimental Affinity (nM)	
					IC50	log10 [IC50]	IC50	log10 [IC50]
A*02:01	5-14	wt	10	KLVVVGAGGV	1,524	3.18	10,914	4.038
	5-14C	C	10	KLVVVGACGV	274	2.43	28,708	4.458
	5-14D	D	10	KLVVVGADGV	762	2.88	19,543	4.291
	5-14R	R	10	KLVVVGARGV	1,573	3.19	20,701	4.316
	5-14V	V	10	KLVVVGAVGV	323	2.51	2,944	3.469
A*03:01	8-16	wt	9	VVGAGGVGK	537	2.73	4,178	3.621
	8-16C	C	9	VVGACGVGK	248	2.39	9,036	3.956
	8-16D	D	9	VVGADGVGK	1,234	3.09	241,546	5.383
	8-16R	R	9	VVGARGVGK	223	2.34	5,297	3.724
	8-16V	V	9	VVGAVGVGK	107	2.03	2,742	3.438
	7-16	wt	10	VVVGAGGVGK	152	2.18	3,428	3.535
	7-16C	C	10	VVVGACGVGK	166	2.22	7,907	3.898
	7-16D	D	10	VVVGADGVGK	409	2.61	47,643	4.678
	7-16R	R	10	VVVGARGVGK	98	1.99	3,236	3.51
	7-16V	V	10	VVVGAVGVGK	120	2.08	3,882	3.589
A*11:01	8-16	wt	9	VVGAGGVGK	111	2.05	817	2.912
	8-16C	C	9	VVGACGVGK	85	1.93	474	2.676
	8-16D	D	9	VVGADGVGK	169	2.22	1,321	3.121
	8-16R	R	9	VVGARGVGK	87	1.93	634	2.802
	8-16V	V	9	VVGAVGVGK	54	1.73	259	2.414
	7-16	wt	10	VVVGAGGVGK	76	2.06	713	2.853
	7-16C	C	10	VVVGACGVGK	99	1.99	536	2.729
	7-16D	D	10	VVVGADGVGK	156	2.2	690	2.839
	7-16R	R	10	VVVGARGVGK	116	2.06	566	2.753
	7-16V	V	10	VVVGAVGVGK	63	1.79	241	2.382
B*07:02	10-19	wt	10	GAGGVGKSAL	2,769	3.44	76,913	4.886
	10-19C	C	10	GACGVGKSAL	4,240	3.62	2,884	3.46
	10-19D	D	10	GADGVGKSAL	5,244	3.72	2,754	3.44
	10-19R	R	10	GARGVGKSAL	70	1.84	44	1.642
	10-19V	V	10	GAVGVGKSAL	2,695	3.43	38,371	4.584

***IC50 Categories for FP-based Assay Procedures**

Affinity	IC50 (nM)	log10 [IC50(nM)]
High	<5,000	<3.7
Medium	5,000-5,0000	3.7-4.7
Low	5,0000-350,000	4.7-5.5
Very Low	350,000-1,000,000	5.5-6
None	>1,000,000	>6

Supplementary Table 2: Predicted and experimental p-HLA stabilities of candidate mKRAS G12 epitopes. Predicted stability measurements were determined using NetMHCstab version 1.0. Experimental stabilities were determined by scintillation proximity assays. Peptide AA sequences are shown with the AA at G12 codon position indicated in *Red*.

HLA Restriction	Peptide ID	G12 AA	Peptide Length	AA Sequence	Predicted Stability (t1/2; hrs)	Experimental Stability (t1/2; hrs)	
						Mean	SD
A*02:01	5-14C	C	10	KLVVVGACGV	3.2	0.8	0.07
	5-14D	D	10	KLVVVGADGV	1.6	0.8	0.07
	5-14R	R	10	KLVVVGARGV	1.8	0.6	0.21
	5-14V	V	10	KLVVVGAVGV	2.9	0.9	0.14
A*03:01	8-16C	C	9	VVGACGVGK	1.8	1.1	0.14
	8-16D	D	9	VVGADGVGK	1.4	0.2	0.07
	8-16R	R	9	VVGARGVGK	2.1	0.6	0
	8-16V	V	9	VVGAVGVGK	2.2	1.2	0
	7-16C	C	10	VVVGACGVGK	1.9	1.2	0.14
	7-16D	D	10	VVVGADGVGK	1.3	0.6	0.07
	7-16R	R	10	VVVGARGVGK	1.8	2.2	0
	7-16V	V	10	VVVGAVGVGK	2.4	2.2	0
A*11:01	8-16C	C	9	VVGACGVGK	2.2	4	0.64
	8-16D	D	9	VVGADGVGK	1.0	0.9	0
	8-16R	R	9	VVGARGVGK	1.0	1.9	0
	8-16V	V	9	VVGAVGVGK	1.8	9.2	0.21
	7-16C	C	10	VVVGACGVGK	4.0	8.3	1.06
	7-16D	D	10	VVVGADGVGK	0.8	6.8	0.14
	7-16R	R	10	VVVGARGVGK	2.2	6	0.14
	7-16V	V	10	VVVGAVGVGK	3.1	12.5	0.42
B*07:02	10-19C	C	10	GACGVGKSAL	0.7	1.3	0.14
	10-19D	D	10	GADGVGKSAL	0.6	0.6	0.21
	10-19R	R	10	GARGVGKSAL	2.1	1.6	0.14
	10-19V	V	10	GAVGVGKSAL	0.8	0.4	0

Supplementary Table 3: Summary of epitopes used as positive controls for biochemical and proteomic studies to validate p-HLA binding. Experimental affinities as determined by competitive peptide binding fluorescence polarization assays and experimental stabilities as determined by scintillation proximity assays are listed. *NA* signifies not applicable.

HLA Restriction	Peptide ID	IEDB ID	Antigen	Organism	AA Sequence	*Experimental Affinity (nM)		Experimental Stability (t1/2; hrs))	
						IC50	log10 [IC50]	Mean	SD
A*02:01	NLV	44920	65 kDa phosphoprotein	Human herpesvirus 5 (Human cytomegalovirus)	NLVPMVATV	1,858	3.269	NA	NA
	gp280-9V	74641	Glycoprotein 100 (anchor-modified)	Homo sapiens (human)	YLEPGPVTV	2,280	3.358	NA	NA
	NA	16650	Poly(A) polymerase catalytic subunit	Vaccinia virus	FLIDLAFLI	NA	NA	10.1	1.27
	NA	54476	Genome polyprotein	West Nile virus	RLARAIIEL	NA	NA	3	0.35
	NA	16979	Latent membrane protein 2	Human herpesvirus 4 (Epstein Barr virus)	FLYALALLL	NA	NA	10	1.84
	NA	121572	Genome polyprotein	Yellow fever virus (Flavivirus febricis)	LLWNGPMAV	NA	NA	5.9	0.21
	NA	69452	55 kDa immediate-early protein 1	Human herpesvirus 5 (Human cytomegalovirus)	VLEETSVML	NA	NA	1.5	1.13
A*03:01	ILR	27283	Nucleoprotein	Influenza A virus	ILRGSVAHK	NA	NA	NA	NA
	NA	65148	POL	Hepatitis B virus	TLWKAGILYK	912	2.96	NA	NA
	gp17-25	2688	Melanocyte protein PMEL	Homo sapiens (human)	ALLAVGATK	962	2.983	NA	NA
	NA	2800	Genome polyprotein	Yellow fever virus (Flavivirus febricis)	ALNTITNLK	NA	NA	10.6	1.2
	NA	110054	Genome polyprotein	Yellow fever virus (Flavivirus febricis)	VLWDIPTPK	NA	NA	4.7	0.28
	NA	70041	Genome polyprotein	Yellow fever virus (Flavivirus febricis)	VMYNLWKMK	NA	NA	0.8	0.07
	NA	232261	Genome polyprotein	Yellow fever virus (Flavivirus febricis)	RVKLSALTLK	NA	NA	12.3	1.06
	NA	232007	Genome polyprotein	Yellow fever virus (Flavivirus febricis)	KLAQRRVFH	NA	NA	3.5	0.07
A*11:01	IVT	29466	Epstein-Barr nuclear antigen 4	Human herpesvirus 4 (Epstein Barr virus)	IVTDFSVIK	NA	NA	NA	NA
	NA	65148	POL	Hepatitis B virus	TLWKAGILYK	1,104	3.043	NA	NA
	gp17-25	2688	Melanocyte protein PMEL	Homo sapiens (human)	ALLAVGATK	225	2.353	NA	NA
	NA	4538	Replicase polyprotein 1ab	Severe acute respiratory syndrome-related coronavirus (Human coronavirus (strain SARS))	ASLPTTIAK	NA	NA	22.5	1.91
	NA	5316	Epstein-Barr nuclear antigen 4	Human herpesvirus 4 (Epstein Barr virus)	AVFDRKSDAK	NA	NA	20.4	0.57
	NA	109407	Serine/threonine-protein kinase PknA	Mycobacterium tuberculosis	KITDFGIAK	NA	NA	15.6	1.48
	NA	67094	Genome polyprotein	Dengue virus	TVNPIVTEK	NA	NA	11.4	0.57
	NA	110054	Genome polyprotein	Yellow fever virus (Flavivirus febricis)	VLWDIPTPK	NA	NA	8	0.64
B*07:02	NA	302918	gp160	human Immunodeficiency virus 1	GRAFVTIGK	371	2.569	NA	NA
	NY60-72	753317	Cancer/testis antigen 1	Homo sapiens (human)	APRGPHGGAASGL	42	1.62	NA	NA
	TPR	65748	65 kDa phosphoprotein	Human herpesvirus 5 (Human cytomegalovirus)	TPRVTGGGAM	NA	NA	2.4	0.21
	NA	232306	Genome polyprotein	Yellow fever virus (Flavivirus febricis)	SPRERLVLTL	NA	NA	1	0.14
	NA	180343	Genome polyprotein	Yellow fever virus (Flavivirus febricis)	RPIDDRFGL	NA	NA	3.2	1.06
	NA	231390	Genome polyprotein	Yellow fever virus (Flavivirus febricis)	RPIDDRFGLAL	NA	NA	1.1	0.07
	NA	232244	Genome polyprotein	Yellow fever virus (Flavivirus febricis)	RPRKTHESHLV	NA	NA	2.4	0.21

***IC50 Categories for FP-based Assay Procedures**

Affinity	IC50 (nM)	log10 [IC50 (nM)]
High	<5,000	<3.7
Medium	5,000-5,0000	3.7-4.7
Low	5,0000-350,000	4.7-5.5
Very Low	350,000-1,000,000	5.5-6
None	>1,000,000	>6

Supplementary Table 4: Characterization of mKRAS tumor cell lines. KRAS gene expression values measured in Transcripts Per Kilobase Million (TPM) for each cell line were determined using the European Bioinformatics Institute (EMBL-EBI) Expression Atlas (https://www.ebi.ac.uk/gxa/home).

Cell Line	KRAS	TPM	Histology	HLA-A	HLA-B	HLA-C
BxPC-3	WT	23	pancreas	01:01 / 01:01	37:01 / 37:01	06:02 / 06:02
RERFLCAD1	G12A	48	lung	11:01 / 24:02	52:01 / 52:01	12:02 / 12:02
NCI-H358	G12C	59	lung	03:01 / 03:01	15:01 / 35:01	03:04 / 04:01
NCI-H2030	G12C	11	lung	11:01 / 24:02	44:03 / 51:01	01:02 / 04:01
PANC1	G12D	18	pancreas	02:01 / 11:01	38:01 / 38:01	12:03 / 12:03
HuCCT1	G12D	17	biliary	11:01 / 11:01	44:02 / 51:01	08:02 / 15:02
CAL-62	G12R	23	thyroid	69:01 / 69:01	35:02 / 44:03	04:01 / 16:01
HuP-T3	G12R	16	pancreas	02:07 / 24:02	40:06 / 40:06	01:02 / 08:01
KP-2	G12R	44	pancreas	24:02 / 26:03	15:01 / 52:01	03:03 / 12:02
PSN1	G12R	130	pancreas	24:02 / 24:02	52:01 / 52:01	12:02 / 12:02
COR-L23	G12V	308	lung	01:01 / 01:01	08:01 / 08:01	07:01 / 07:01
NCI-H441	G12V	92	lung	02:02 / 03:01	38:01 / 44:03	16:02 / 16:02
QGP-1	G12V	110	pancreas	24:02 / 24:02	15:11 / 67:02	01:21 / 12:03
SK-CO-1	G12V	56	colon	01:01 / 02:01	35:08 / 58:05	05:01 / 07:01
SW620	G12V	78	colon	02:01 / 24:02	07:13 / 37:04	07:04 / 07:04
YAPC	G12V	107	pancreas	24:02 / 24:02	35:01 / 52:01	12:02 / 12:02

Supplementary References

1. Buchli, R. *et al.* Development and validation of a fluorescence polarization-based competitive peptide-binding assay for HLA-A*0201--a new tool for epitope discovery. *Biochemistry* **44**, 12491–507 (2005).
2. Pino, L. K. *et al.* The Skyline ecosystem: Informatics for quantitative mass spectrometry proteomics. *Mass Spectrom. Rev.* **39**, 229–244 (2020).
