# Supplementary material for: Biochemical and functional characterization of mutant KRAS epitopes validates this oncoprotein for immunological targeting
Source: Nat Commun. 2021 Jul 16;12:4365. doi: 10.1038/s41467-021-24562-2 (PMC8285372; doi:10.1038/s41467-021-24562-2)
Supplement: Supplementary file 3 — Descriptions of additional Supplementary Files [file 41467_2021_24562_MOESM3_ESM.pdf]

Descriptions of Additional Supplementary Files

Supplementary Data 1
Description: Neoantigen prediction tool antigen.garnish output file.

Supplementary Movie 1
Description: Time lapse video monitoring over 150h demonstrating (a) TCRA3V cell specific killing of CORL23-A3 cells and (b)TCRA11V cell specific killing of CORL23-A11 cells plated at E:T ratio of 3:1. Tumor cells are marked with GFP via modification with the HLA-SCD construct and appear green. The media was supplemented with Annexin V-CF594 to identify apoptotic cells which appear red.
