# Supplementary figures and images for: Biochemical and functional characterization of mutant KRAS epitopes validates this oncoprotein for immunological targeting
Source: Nat Commun. 2021 Jul 16;12:4365. doi: 10.1038/s41467-021-24562-2 (PMC8285372; doi:10.1038/s41467-021-24562-2)

## Slide 1
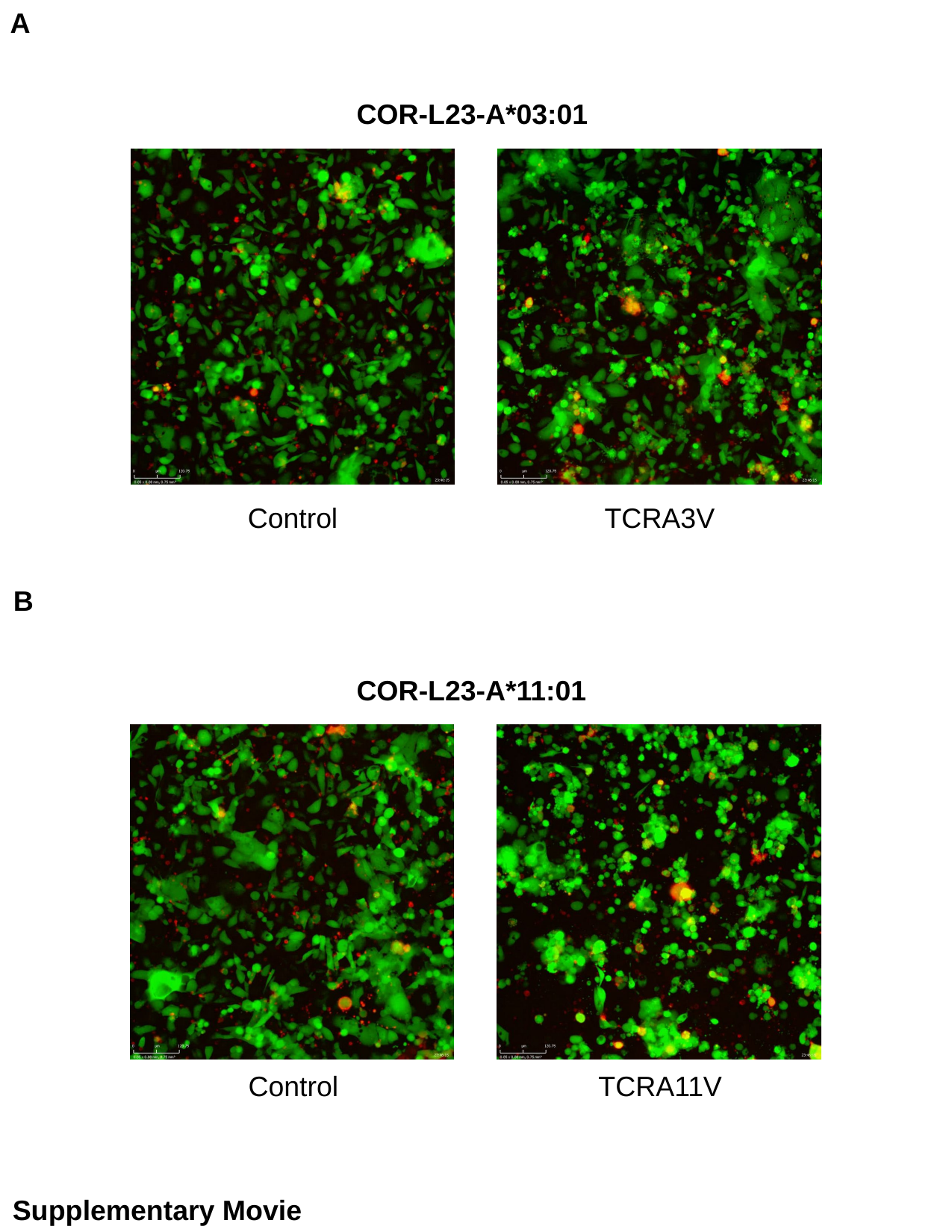

A
COR-L23-A*03:01
Control
TCRA3V
B
COR-L23-A*11:01
Control
TCRA11V
Supplementary Movie

Supplement: Supplementary file 5 — Supplementary Movie 1 [file 41467_2021_24562_MOESM5_ESM.pptx]
